# RETRACTED: Kim et al. Autocrine Motility Factor and Its Peptide Derivative Inhibit Triple-Negative Breast Cancer by Regulating Wound Repair, Survival, and Drug Efflux. *Int. J. Mol. Sci.* 2024, *25*, 11714

**DOI:** 10.3390/ijms27219824

**Published:** 2026-11-02

**Authors:** Se Gie Kim, Seok Joong Kim, Thanh Van Duong, Yuhan Cho, Bogeun Park, Ulhas Sopanrao Kadam, Hee Sung Park, Jong Chan Hong

**Affiliations:** 1 Department of Cosmetic Science, Kyungsung University, Busan 48434, Republic of Korea; 2 Department of Food and Nutrition, College of Natural and Information Science, Dongduk Women’s University, Seoul 02758, Republic of Korea; 3 Department of Anatomy, School of Medicine, Pusan National University, Yangsan 50612, Republic of Korea; 4 Division of Applied Life Science (BK21 Four), Plant Molecular Biology and Biotechnology Research Center, Gyeongsang National University, Jinju 52828, Republic of Korea; yoohan2138@gmail.com (Y.C.); ulhasskadam@gmail.com (U.S.K.)

The journal retracts the article titled “Autocrine Motility Factor and Its Peptide Derivative Inhibit Triple-Negative Breast Cancer by Regulating Wound Repair, Survival, and Drug Efflux” [1], cited above.

Following the publication, concerns were brought to the attention of the Editorial Office regarding the image and data integrity issues presented in this paper [1].

In accordance with the journal’s standard procedures, the Editorial Office and Editorial Board conducted an investigation, which confirmed that a nonspecific anti-Bax antibody (sc-7480) had been used, making the reported Bax protein expression data unreliable. Moreover, the investigation identified partial image duplication within Figure 3, and between Figure 5 and Figure 6 of this publication [1].

Although the authors cooperated with the investigation, the explanations and supporting materials provided were not deemed sufficient by the Editorial Board to resolve the identified concerns. As a result, the Editorial Board has decided to retract the article in line with the MDPI retraction policy (https://www.mdpi.com/ethics#_bookmark30).

This retraction was approved by the Editor-in-Chief of the journal *International Journal of Molecular Sciences*.

The authors have been informed of this retraction and have indicated their agreement with this decision.
